# Supplementary figures and images for: Structural insight into the dual function of LbpB in mediating Neisserial pathogenesis
Source: eLife. 2021 Nov 9;10:e71683. doi: 10.7554/eLife.71683 (PMC8577839; doi:10.7554/eLife.71683)

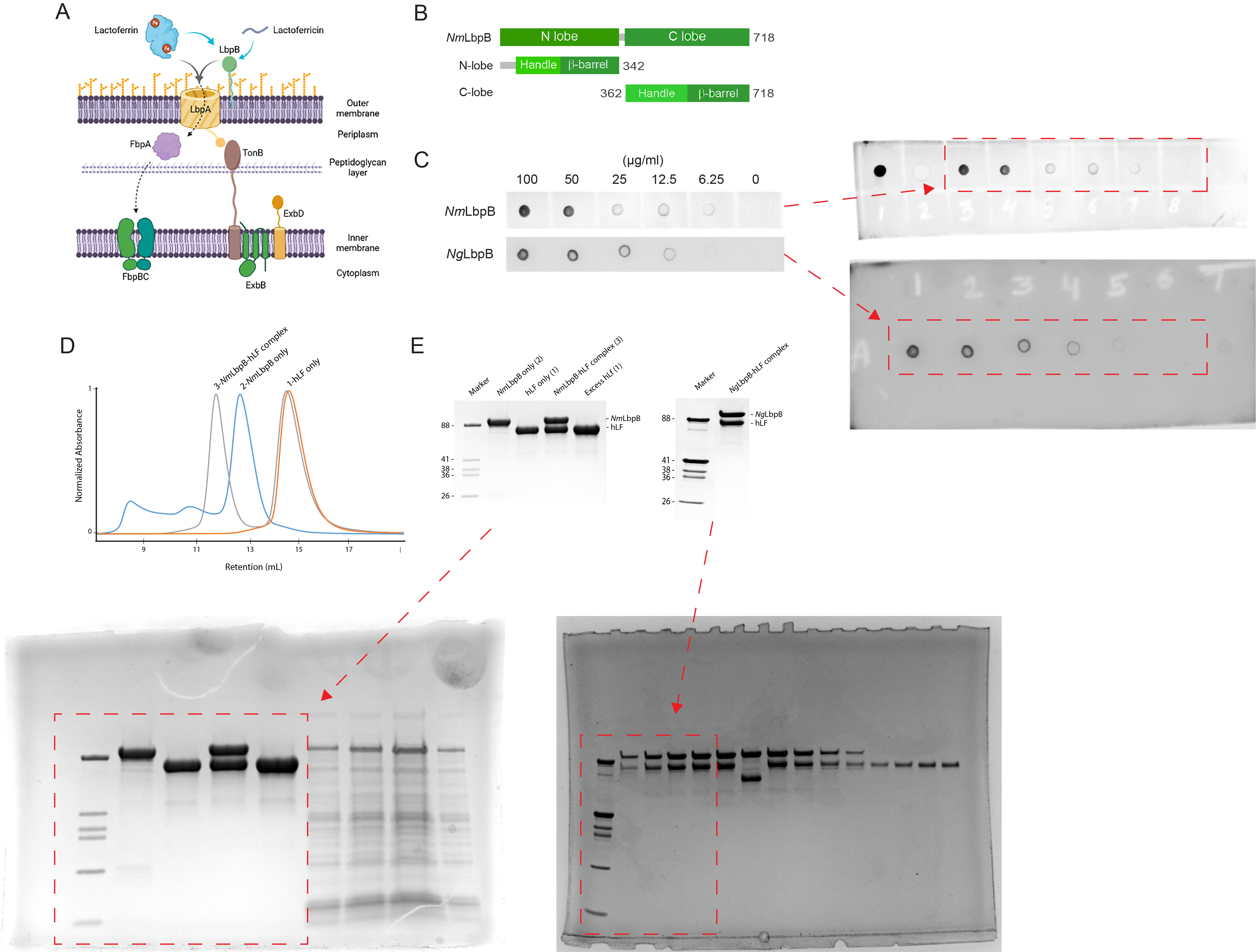

Supplement: Figure 1—source data 1. — (A) The proposed role of lactoferrin-binding protein (Lbp) system in iron acquisition from lactoferrin and protection from lactoferricin (Biorender). (B) Summary of LbpB constructs used in this study. (C) Solid-phase-binding assay of holo-lactoferrin (Lf) binding to N. meningitidis LbpB (NmLbpB; anti-Lf) and N. gonorrhoeae LbpB (NgLbpB; Lf-horse radish peroxidase [HRP]). (The red arrow indicates the original blots that werecropped for this panel.) (D) Formation of the NmLbpB–Lf complex over size-exclusion chromatography (SEC) from purified components. A leftward shift is observed for the complex compared to the individual components indicating the formation of the complex. (E) Sodium dodecyl sulphate-polyacrylamide gel electrophoresis (SDS–PAGE) analysis of the NmLbpB–Lf complex formed from panel D, indicating the formation of the complex at a 1:1 ratio (lane 4). Similarly, the NgLbpB–Lf complex was formed by SEC from purified components which alsoformed a 1:1 complex as shown by SDS–PAGE analysis. (The red arrow indicates the original SDS–PAGE gels that were cropped for this panel.) [file elife-71683-fig1-data1.jpg]

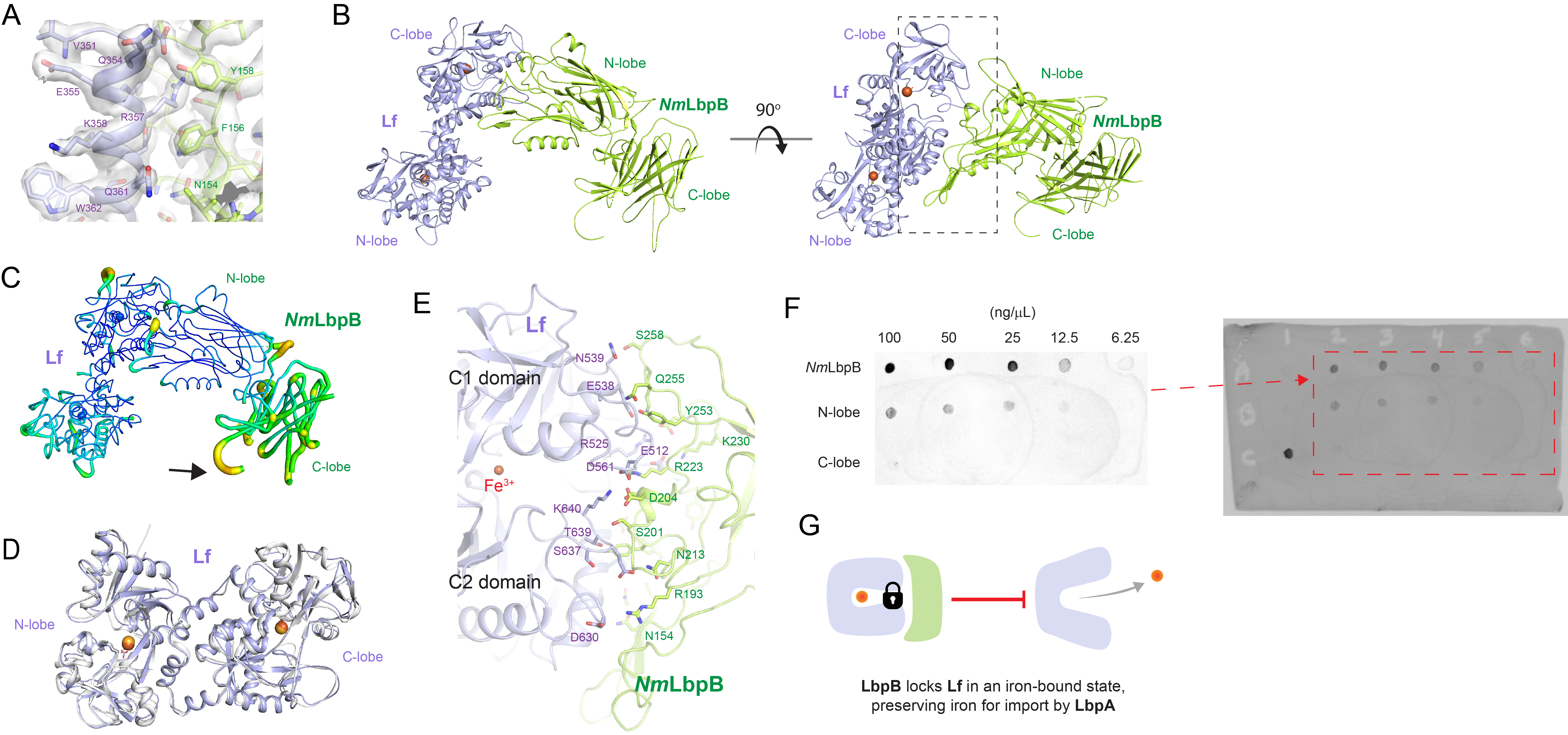

Supplement: Figure 3—source data 1. — (A) Zoomed view at the interface between NmLbpB and lactoferrin (Lf) depicting the quality of the electron density shown as a grey isosurface (2FO–FC , 1.0σ). (B) Orthogonal views of the complex with NmLbpB in green, Lf in violet, and the iron atoms as red spheres. The N-lobe of NmLbpB interacts with only the C-lobe of Lf along an extended interface. (C) The C-lobe of NmLbpB hashigh B-factors with the large loops of this lobe not observed in our structure; the black arrow indicates the putative location of these loops. (D) An alignment of Lf from the complex with the structure of uncomplexed Lf (PDB ID 2BJJ) shows very little conformational changes upon binding NmLbpB (RMSD of 1.3 Å). (E) A zoomed view of the binding interface shows extensive interactions along an elongated surface covering both the C1 and C2 domains of Lf (buried surface area 1760.8 Å 2 ). (F) Solid-phase-binding assays show Lf binds both full-length and N-lobe NmLbpB, but not C-lobe only, supporting the observations in the complex structure. (The red arrow indicates the original blot that was cropped for this panel.) (G) Much like what has been proposed for the role of transferrin-binding protein (Tbp) B in the Tbp system, here we propose that LbpB also serves to bind and lock Lf in an iron-bound state for delivery to LbpA for iron import. [file elife-71683-fig3-data1.jpg]

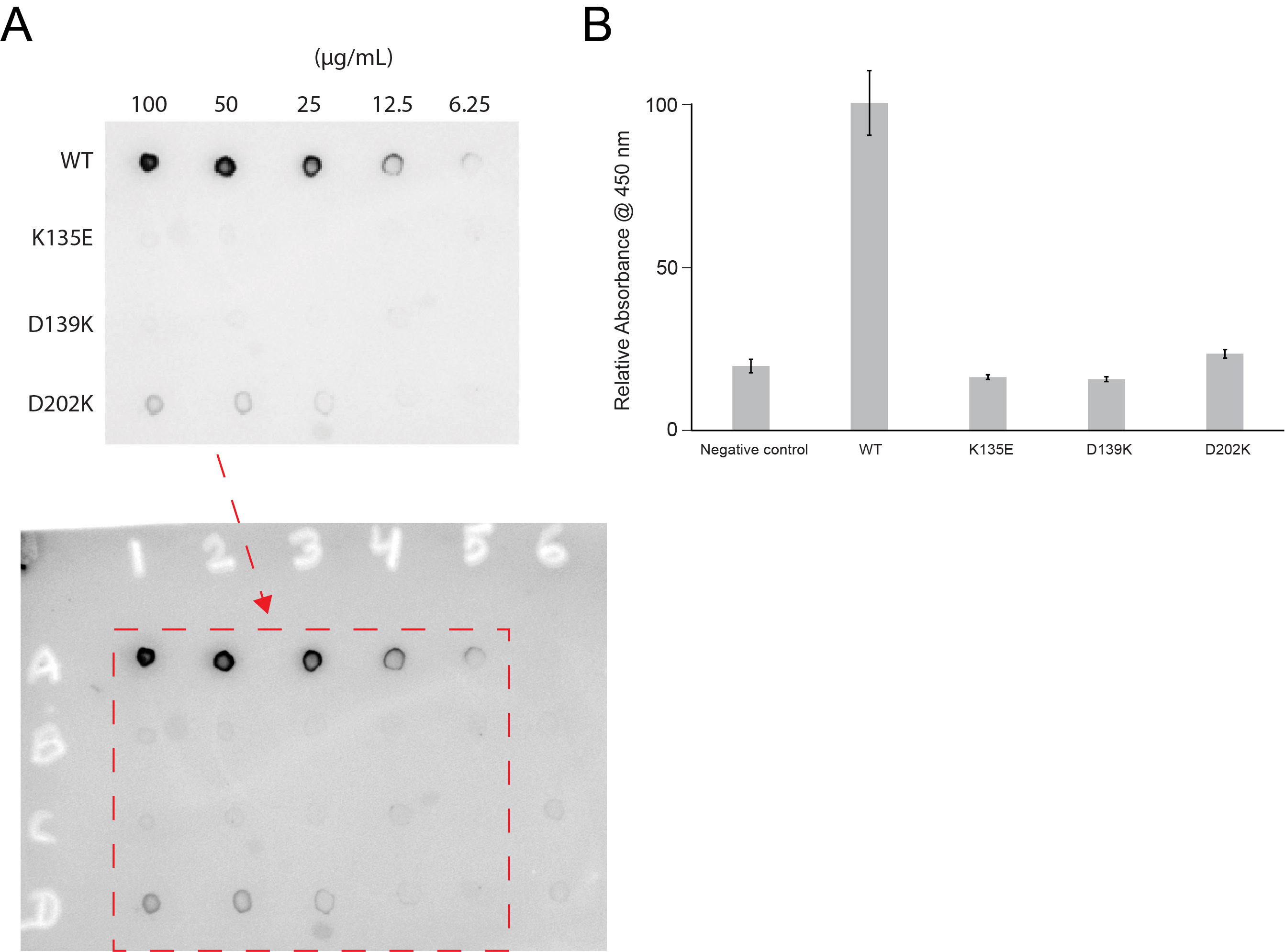

Supplement: Figure 4—figure supplement 2—source data 1. — (A) Solid-phase-binding assays ofdilutions of NgLbpB and mutants with horse radish peroxidase (HRP)-conjugated lactoferrin probe. (The red arrow indicates the original blot that was cropped for this panel.) (B) Enzyme-linked immunosorbent assays (ELISAs) of NgLbpB andmutants showing normalized absorbance. All experiments were done at least in triplicate. [file elife-71683-fig4-figsupp2-data1.jpg]

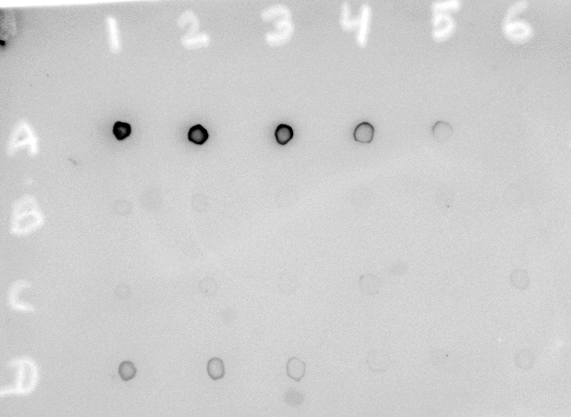

Supplement: Source data 1. [file elife-71683-supp2.zip › yadav_source_files_v2/Figure 4-figure supplement 2_panelA_source.tif]

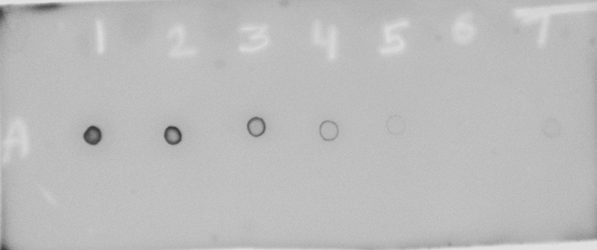

Supplement: Source data 1. [file elife-71683-supp2.zip › yadav_source_files_v2/Figure1_panelC_2_source.tif]
