# Supplementary material for: Structural insight into the dual function of LbpB in mediating Neisserial pathogenesis
Source: eLife. 2021 Nov 9;10:e71683. doi: 10.7554/eLife.71683 (PMC8577839; doi:10.7554/eLife.71683)
Supplement: Supplementary file 1. — (a) Summary of size-exclusion chromatography small-angle X-ray scattering (SEC-SAXS) parameters. Lactoferrin (Lf) has a calculated molecular weight of 76.3 kDa, lactoferricin (Lfcn) 1.4 kDa, N. gonorrhoeae LbpB (NgLbpB) 78.4 kDa, and N. meningitidis LbpB (NmLbpB) 79.5 kDa. (b) Data collection and refinement statistics for the NmLbpB–Lf X-ray crystal structure. (c) Data collection and refinement statistics for the NgLbpB–Lf cryoEM structure. (d) Summary of the intermolecular interactions between NmLbpB and Lf. The information about interacting residues was obtained by QtPISA analysis. (e) Intermolecular interactions between NgLbpB and Lf. The information about interacting residues was obtained by QtPISA analysis. (f) Summary of ITC parameters for lactoferrin binding to NmLbpB mutants. These experiments were performed using a MicroCal iTC200 ITC calorimeter (Malvern Panalytical). (g) Summary of ITC parameters for lactoferricin binding to NmLbpB loop deletions. These experiments were performed using a Nano ITC calorimeter (TA Instruments). (h) Summary of ITC parameters for lactoferrin and lactoferricin binding to NmLbpB. These experiments were performed using a Nano ITC calorimeter (TA Instruments). [file elife-71683-supp1.docx]

**Supplementary File 1.­­**

**Supplementary File 1a. Summary of SEC-SAXS parameters.** Lactoferrin (Lf) has a calculated molecular weight of 76.3 kDa, lactoferricin (Lfcn) 1.4 kDa, *Ng*LbpB 78.4 kDa, and *Nm*LbpB 79.5 kDa.

|  | **Lf** | ***Ng*LbpB** | | | ***Nm*LbpB** | | | |
| --- | --- | --- | --- | --- | --- | --- | --- | --- |
|  |  |  | **+Lf** | **+Lfcn** |  | **+Lf** | **+Lfcn** | **+Lf+Lfcn** |
| R_g_ | 33.2 ± 0.3 | 37.8 ± 0.1 | 45.9 ± 0.1 | 37.7 ± 0.2 | 35.9 ± 0.1 | 44.6 ± 0.2 | 34.9 ± 0.2 | 44.6 ± 0.1 |
| V_c_ MW | 75.8 | 88.8 | 168.0 | 66.8 | 88.9 | 155.3 | 83.7 | 171.3 |
| V_p_ MW | 88.1 | 106.3 | 190.9 | 86.3 | 104.6 | 178.8 | 98.2 | 208.7 |
| Bayes MW | 80.8 | 94.2 | 169.6 | 74.3 | 94.2 | 157.1 | 85.7 | 185.8 |
| Shape/Size MW | 81.4 | 102.2 | 181.9 | 89.9 | 98.3 | 150.0 | 86.8 | 202.2 |
| D_max_ | 110 | 114 | 139 | 119 | 116 | 134 | 117 | 135 |

**Supplementary File 1b. Data collection and refinement statistics for the *Nm*LbpB-Lf X-ray crystal structure.**

| **Data Collection** | ***Nm*LbpB-Lf** |
| --- | --- |
| λ (Å) | 1.0 |
| Space group | P 4_3_2_1_2 |
| a, b, c (Å) | 120.39, 120.39, 207.38 |
| α, β, γ (º) | 90, 90, 90 |
| Resolution (Å)^*^ | 50 – 2.85 (2.95 – 2.85) |
| Completeness (%)^*^ | 99.9 (100) |
| Redundancy^*^ | 8.9 (8.7) |
| R_sym_ ^†*^ | 0.263 (2.93) |
| I / σ (I)^*^ | 14 (1.33) |
| CC_1/2_ | 0.985 (0.405) |
| **Refinement** |  |
| Resolution (Å) | 47.62 – 2.85 (2.95 – 2.85) |
| No. reflections | 36,134 (3516) |
| R^§^/R_free_^¶^ | 0.20/0.25 |
| **r.m.s. deviations** |  |
| Bonds (Å) | 0.002 |
| Angles (º) | 0.57 |
| No. Protein atoms | 9,459 |
| No. Ligand atoms | 75 |
| No. Waters | 13 |
| **B-factors** (Å^2^) |  |
| Wilson B | 77.46 |
| Protein | 87.41 |
| Ligands | 133.38 |
| Waters | 60.17 |
| **Ramachandran Analysis^¥^** |  |
| Favored (%) | 92.58 |
| Allowed (%) | 7.18 |
| Outliers (%) | 0.24 |
| PDB ID | 7JRD |

^†^*R*_sym_ = Σ*_hkl,j_* (|I_hkl_-<I_hkl_>|) / Σ*_hkl,j_* I_hkl_, where <I_hkl_> is the average intensity for a set of j symmetry related reflections and I_hkl_ is the value of the intensity for a single reflection within a set of symmetry-related reflections.

^§^ *R* factor = Σ*_hkl_* (||F_o_| - |F_c_||) / Σ*_hkl_*_|_F_o_| where F_o_ is the observed structure factor amplitude and F_c_ is the calculated structure factor amplitude.

^¶^ *R*_free_ = Σ*_hkl,T_* (||F_o_| - |*F*_c_||) / Σ*_hkl,T_*|*F*_o_|, where a test set, T (5% of the data), is omitted from the refinement.

^¥^ Performed using Molprobity within PHENIX.

^*^ Indicates statistics for last resolution shell shown in parenthesis.

**Supplementary File 1c. Data collection and refinement statistics for the *Ng*LbpB-Lf cryoEM structure.**

|  | *Ng*LbpB-Lf |
| --- | --- |
| **Data Collection and Processing** | |
| Magnification | 81,000 |
| Voltage (kV) | 300 |
| Electron exposure (e^-^/Å^2^) | 53.68 |
| Defocus range (µm) | -1 to -2.5 |
| Pixel size (Å) | 0.54 |
| Symmetry imposed | C1 |
| Initial particle projections (no.) | 3,330,059 |
| Final particle projections (no.) | 127,832 |
| Map resolution (Å) | 3.65 |
| FSC threshold | 0.143 |
| **Refinement** | |
| Model Resolution (Å) | 3.9 |
| FSC threshold | 0.5 |
| Map-model CC |  |
| CC_mask | 0.72 |
| CC_box | 0.74 |
| CC_peaks | 0.67 |
| CC_volume | 0.72 |
| Model Composition |  |
| Non-hydrogen atoms | 9405 |
| Protein residues | 1220 |
| Ligands | 4 |
| B factors (Å^2^) |  |
| Protein | 72.19 |
| Ligand | 82.43 |
| R.M.S. deviations |  |
| Bond lengths (Å) | 0.008 |
| Bond angles (°) | 1.020 |
| Validation |  |
| MolProbity Score | 2.70 |
| Clashscore | 35.26 |
| Rotamer outliers (%) | 0.81 |
| Ramachandran Plot |  |
| Favored (%) | 78.39 |
| Allowed (%) | 21.36 |
| Outliers (%) | 0.25 |
| PDB | 7N88 |
| EMDB | EMD-24233 |

**Supplementary File 1d. Summary of the intermolecular interactions between *Nm*LbpB and Lf.** The information about interacting residues was obtained by QtPISA analysis.

| ***Nm*LbpB** | **Lf** | **Distance** |
| --- | --- | --- |
| **Hydrogen bonds** | | |
| R135 | R357 | 2.8 |
| R135 | Q513 | 2.9 |
| A136 | R357 | 2.9 |
| K139 | R357 | 2.6 |
| N141 | R357 | 3.5 |
| N154 | N360 | 3.6 |
| Y158 | E353 | 2.2 |
| R193 | D630 | 2.7 |
| S201 | S637 | 2.9 |
| S201 | T639 | 3.9 |
| T203 | K640 | 3.6 |
| D204 | D561 | 3.5 |
| Y210 | Q354 | 3.0 |
| Y210 | T639 | 3.9 |
| N213 | E638 | 2.8 |
| Y220 | Q513 | 2.5 |
| K230 | E512 | 3.5 |
| Y253 | E538 | 2.6 |
| Q255 | A537 | 3.4 |
| K257 | E538 | 3.6 |
| S258 | N539 | 3.0 |

| ***Nm*LbpB** | **Lf** | **Distance** |
| --- | --- | --- |
|  | **Salt bridges** |  |
| K117 [NZ] | E515 [OE1] | 3.4 |
| R135 [NE] | E353 [OE1] | 3.7 |
| R135 [NH1] | D511 [OD2] | 3.6 |
| R135 [NH2] | D511 [OD1] | 4.0 |
| R135 [NH2] | D511 [OD2] | 3.8 |
| D204 [OD1} | K640 [NZ] | 3.0 |
| D204 [OD2] | K640 [NZ] | 3.5 |
| R223 [NE] | D561 [OD2] | 3.9 |
| R223 [NH2] | D561 [OD2] | 3.6 |
| D227 [OD1] | R525 [NH1] | 3.5 |
| D227 [OD2] | R525 [NH1] | 2.8 |
| D227 [OD2] | R525 [NH2] | 3.4 |
| K230 [NZ] | E512 [OE1] | 2.9 |
| K257 [NZ] | E538 [OE1] | 3.1 |
| K257 [NZ] | E538 [OE2] | 3.5 |

**Supplementary File 1e. Intermolecular interactions between *Ng*LbpB and Lf.** The information about interacting residues was obtained by QtPISA analysis.

| ***Ng*LbpB** | **Lf** | **Distance** |
| --- | --- | --- |
| **Hydrogen bonds** | | |
| E118 [OE1] | Q513 [NE2] | 3.8 |
| E118 [OE2] | Q513 [NE2] | 3.9 |
| Y131 [OH] | Q513 [NE2] | 3.2 |
| G137 [O] | R357 [NH1] | 2.3 |
| D139 [OD2] | N360 [ND2] | 2.3 |
| R191 [NH2] | D630 [O] | 2.7 |
| S200 [N] | E638 [O] | 3.4 |
| D202 [OD1] | N560 [N] | 3.7 |

| ***Ng*LbpB** | **Lf** | **Distance** |
| --- | --- | --- |
| **Salt bridges** | | |
| R113 [NH1] | D511 [OD2] | 3.9 |
| R113 [NH1] | E515 [OE1] | 3.5 |
| R113 [NH1] | E515 [OE2] | 3.8 |
| K117 [NZ] | E515 [OE2] | 3.7 |
| K228 [NZ] | E512 [OE1] | 3.4 |

**Supplementary File 1f. Summary of ITC parameters for lactoferrin binding to *Nm*LbpB mutants.** These experiments were performed using a MicroCal iTC200 ITC calorimeter (Malvern Panalytical).

*NmLbpB interface mutants*

| Protein | K_d_ (μM) | ΔH (kcal/mol) | ΔS (kcal/mol/deg) | n |
| --- | --- | --- | --- | --- |
| Wt | 0.1 ± 0.1 | -15.8 ± 2.5 | -0.02 ± 0.01 | 0.8 ± 0.1 |
| R193E | 2.0 ± 0.2 | -11.8 ± 0.3 | -0.014 ± 0.01 | 0.75 ± 0.04 |
| D227K | 1.9 ± 0.6 | -5.4 ± 0.3 | 0.008 ± 0.002 | 0.7 ± 0.1 |
| R135E | 11.5 ± 1.3 | -9.7 ± 1.1 | -0.02 ± 0.01 | 0.9 ± 0.1 |
| D140K | 0.9 ± 0.01 | -9.5 ± 0.1 | -0.004 ± 0.0004 | 0.6 ± 0.01 |
| K143E | 0.3 ± 0.2 | -16.7 ± 0.2 | -0.03 ± 0.01 | 0.6 ± 0.04 |

**Supplementary File 1g. Summary of ITC parameters for lactoferricin binding to *Nm*LbpB loop deletions.** These experiments were performed using a Nano ITC calorimeter (TA Instruments).

*NmLbpB loop deletions*

| Construct | K_d_ (µM) | ΔH (kcal/mol) | ΔS (cal/mol.K) | n |
| --- | --- | --- | --- | --- |
| WT | 3.7 | -11.4 ± 0.5 | -15.5 | 1.3 ± 0.03 |
| Δ372-383 | 4.9 | -9.9 ± 1.0 | -10.8 | 1.4 ± 0.1 |
| Δ416-418 | 5.9 | -9.1 ± 0.6 | -8.1 | 1.6 ± 0.1 |
| Δ445-526 | 9.1 | -9.6 ± 2.0 | -10.9 | 0.9 ± 0.1 |
| Δ561-565 | 4.1 | -10.1 ± 0.5 | -11.1 | 1.3 ± 0.04 |
| Δ594-599 | 4.8 | -9.7 ± 0.8 | -10.0 | 1.6 ± 0.1 |
| Δ665-698 | 5.6 | -10.7 ± 0.7 | -13.8 | 1.5 ± 0.1 |
| Δ445-526_ Δ665-698 | insufficient binding to determine accurately | | | |

**Supplementary File 1h. Summary of ITC parameters for lactoferrin and lactoferricin binding to *Nm*LbpB.** These experiments were performed using a Nano ITC calorimeter (TA Instruments).

*Titration with Lactoferrin (Lf)*

| Constructs | K_d_ (µM) | ΔH (kcal/mol) | ΔS (cal/mol.K) | n |
| --- | --- | --- | --- | --- |
| *Nm*LbpB | 0.45 | -15.1 ± 0.3 | -21.7 | 0.7 ± 0.01 |
| *Nm*LbpB-Lfcn | 0.45 | -14.6 ± 0.4 | -20.0 | 0.8 ± 0.01 |

*Titration with lactoferricin (Lfcn)*

| Construct | K_d_ (µM) | ΔH (kcal/mol) | ΔS (cal/mol.K) | n |
| --- | --- | --- | --- | --- |
| *Nm*LbpB-Lf | 7.29 | -10.6 ± 1.3 | -14.0 | 1.4 ± 0.1 |
